# Victimhood and Blame Dialectics in Culturally Diverse Male Students’ Discussions About Sexual Assault Policies

**DOI:** 10.1177/10608265231182100

**Published:** 2023-06-04

**Authors:** KelleyAnne Malinen, Brooke VanTassel, Karen Kennedy, Emily MacLeod, Kristin O'Rourke

**Affiliations:** 1 444899 Mount Saint Vincent University Department of Sociology and Anthropology, Halifax, NS, Canada; 2 176726 Halifax Regional Centre for Education, Dartmouth, NS, Canada; 3 Department of Nursing, 55964 Cape Breton University, Sydney, NS, Canada; 4 Department of Education, 55964 Cape Breton University, Sydney, NS, Canada

**Keywords:** sexual violence policy, male victims, false allegations, dialectics, cultural diversity

## Abstract

Culture and Perspectives on Sexual Assault Policy was a qualitative, focus-group study conducted at four Canadian universities to gather culturally diverse student perspectives on university sexual violence or sexual assault policies and services. This article highlights two categories of dialectical tension expressed during several male focus groups. The Wrongful Blame Dialectic involved tension between anxieties about wrongful accusations and opposition to victim-blaming. Perceived risk of wrongful accusations was often linked to racism or ethnocentrism. The Male Victim Denial/Recognition Dialectic involved tension between denial and recognition of male sexual victimization. Male participants felt more vulnerable to wrongful accusation than to sexual violence. They felt more likely to be blamed and disbelieved, whether as respondents or complainants.

Perspectives of male-identified (henceforth, male) students regarding university sexual violence or sexual assault policies (henceforth SV/SA policies) are an essential yet understudied aspect of how SV/SA measures may be used and experienced by students. Male students comprise a culturally diverse group with overlapping and distinct views. This article presents qualitative responses of culturally diverse male focus group participants to their SV/SA policies, focusing on two dialectical tensions.

Experiences of sexual violence and related services are shaped by individuals’ lives (Worthen & Wallace, 2017), including intersectional identities such as gender, race, and culture. While challenging to define (Kroeber & Kluckhohn, 1952), culture is generally understood as a multifactorial construct formed over a long time within a group or community (Taras et al., 2009). Definitions, perceptions, experiences, and consequences of SV/SA vary culturally (Boy & Kulczycki, 2008; Chan, 2009; Dussich, 2001; Equality Now & Dignity Alliance International, 2021; Ilkkaracan, 2015; Ling, 2007). So do normative repertoires of gender (Connell, 2005) and, according to our data, students’ expectations and experiences of postsecondary policies and practices. Therefore, equitable SV/SA responses should be customized to serve a range of ethnic groups or identities based on their languages, histories, and historical marginalization. We developed our study to answer the intersectional question: “How can Nova Scotia universities build capacity for culturally sensitive sexual violence prevention and response that also accounts for gender identity?”

Sexual violence or sexual assault (SV/SA) policies at our institutions include definitions of sexual violence and/or sexual assault, avenues for investigation, possibilities for sanction, safety measures, confidentiality rules, and supports. We convened forty-two focus groups representing 14 cultural communities at four universities between 2018 and 2021 in Nova Scotia, Canada. Participants learned about and responded to their university SV/SA policies. Each group was matched for gender identity (male, female, or transgender/non-binary) and cultural identity. Region or country of origin served as a proxy for culture. Constructivist grounded theory analysis of transcripts surfaced dialectical tensions between participants as well as within the narratives of individuals, leading us to take up dialectics as a theoretical touchstone and sensitizing concept in analysis (Baxter & Montgomery, 1996).

Male students from diverse cultural communities navigated dialectical tensions that did not emerge within other gender groups. The first tension involved fears about wrongful accusations on the one hand, and concerns about victim-blaming on the other. As part of this theme, men of African descent feared racial bias would generate or facilitate false accusations or prejudicial investigations. The second tension involved denial versus recognition of male sexual victimization. Thus, the two themes presented here are the “Wrongful Blame Dialectic” and the “Male Victim Denial/Recognition Dialectic.” This article is intended to support increased equity by prompting dialectical engagement between SV/SA policy makers and service providers and the anxieties of culturally diverse male participants.

## Context

The 2010s were punctuated in our province and across Canada by mediatized campus SV incidents (Backhouse et al., 2015; Haiven, 2017; Mahon, 2014; Pace, 2017) generating a push for university SV/SA policies. Part of this effort was a document released by the Province of Nova Scotia & Council of Nova Scotia University Presidents (2017), *Guidelines and Recommendations for Nova Scotia Universities and the Nova Scotia Community College: Development of survivor-centric sexual violence policies and responses*. This document mandates maintenance of “up to date stand-alone sexual violence policies that are survivor-centric” (p. 3). SV/SA policies outline how campus community members can pursue support or justice following SV/SA incidents. Supports can typically be sought with or without requesting an investigation be conducted. In all policies reviewed for this study, terms like victim, survivor, complainant, and respondent were gender neutral.

Understanding cultural differences among students is essential in developing university SV/SA policies. The provincial government’s *Guidelines and Recommendations for Nova Scotia Universities* document highlights culture’s importance to SV/SA response without indicating how to achieve cultural responsiveness in this context. US research on students’ perspectives regarding SV usually deploys culturally undifferentiated samples (Romero-Sánchez & Megías, 2015). The same tends to apply in Canada. International research outside postsecondary environments shows victim-blaming and stigmatization are prevalent worldwide, with cultural and national variation in how SV is perpetrated, defined, perceived, and responded to (Boy & Kulczycki, 2008; Chan, 2009; Contreras et al., 2010; Dussich, 2001; Equality Now & Dignity Alliance International, 2021; Ilkkaracan, 2015; Ling, 2007). In colonial societies, women of colour are less likely than white women to be seen as legitimate SV/SA victims, whereas men of colour have often been stereotyped as dangerous predators (Butler, 1993; Crenshaw, 1991; Davis, 1983; Duru, 2004; Fanon, 2008; Maynard, 2017) – facts that should be considered in SV/SA policy and service development.

## Literature Review

### Dialectics, Culture, and Sexual Violence

Dialectics, “the art of thinking the coincidence of distinctions and connections” (Bhaskar, 2008, p. 180; see also Gunnarsson, 2018, p. 5; Semlak et al., 2008), is useful when considering SV/SA, a domain replete with dichotomies such as privacy/disclosure, masculine/feminine, victim/perpetrator, consensual/non-consensual, virgin/whore, and powerful/powerless (Gunnarsson, 2018; Wright, 2022). This approach has Eastern and Western roots, although it may come more naturally to those steeped in Eastern traditions (e.g., Dafermos, 2018; Peng & Nisbitt, 1999; Lister, 1997). Dialectics are applied to many forms of social research, including intercultural communications (Martin & Nakayama, 1999; Rodriguez, 2002; Cheong & Gray, 2011; Dutta-Bergman, 2004). Interactions related to SV/SA policies can be high stakes intercultural engagements for the students affected by them, involving micro and macro power relations (Curtin, 2013; Halualani & Nakayami, 2013). Power relations unfold among 1) those who have experienced, disclosed, reported, or remained silent about SV/SA; 2) those who have perpetrated or allegedly perpetrated it; and 3) responding institutional representatives. The Wrongful Blame Dialectic and the Male Victim Denial/Recognition Dialectic relate to activist and scholarly tensions in this area. For example, Maricourt and Burrell (2022) associate anxieties about wrongful allegations with anti-feminist backlash. Gotell and Dutton’s (2016) analysis of Canadian Men’s Rights websites identifies exaggerating the likelihood of false allegations and gender-neutral framings of SV/SA as backlash strategies.

### Wrongful Blame Dialectic

As stated by Lisak and colleagues (2010) “within the domain of rape, the most highly charged area of debate concerns the issue of false allegations” (p. 1318). Dialectical tension prevails between those who believe discussing wrongful allegations constitutes anti-feminist backlash (Gotell & Dutton, 2016; Lonergan, 2018) and those who view false allegations as a possibility legislation and policies must protect against. Gender figures prominently in this domain. Reflective of this tension, one study found nearly all of a sample claiming to be wrongfully accused of adult SA were men (Rumney & McCartan, 2017). Another study found female gendered language was used by police discussing false reporting (Lisak et al., 2010).

Researchers who address wrongful allegations often affirm the view shared by feminists that such allegations are rare and that their frequency is exaggerated in victim-blaming discourse and rape mythology (Rumney & McCartan, 2017; Lisak et al., 2010, p. 1321). Crucially, when they do occur, false accusations are often unintentional (Engle & O’Donohue, 2012). Thus, Rumney and McCartan (2017) do not advocate criminalizing false complaints. Nonetheless, wrongful allegations, however unusual and unintentional, can result in “besmirched reputations, interruptions in important life functions and, in some cases, incarceration” (Engle & O’Donohue, 2012, p. 101).

The origin of the frequently cited two percent false allegation rate is unclear. By reviewing research, Lisak and colleagues (2010) found the actual number falls between two and ten percent. The same researchers found six percent of SA cases reported to a US university police department over ten years were false. After reviewing police files, they categorized a case as a false only if “thorough investigation” had generated clear evidence that the reported offense “had in fact not occurred” (p. 1328). Individuals who feel at risk for wrongful accusations, particularly members of disproportionately policed groups, may not be reassured by the fact that at least nine out of ten complaints appear to be legitimate.

In white supremacist cultures, Blackness has been attributed to danger and criminalized. In Canada, Black people have endured intense police surveillance impacting their quality of life (Maynard, 2017). One form of stereotyping, rooted in dehumanizing justifications for slavery, has viewed Black men as “hypersexual, animalistic and violent,” oppositional and threatening to white identity (Ferber, 2007, p. 14). As Ferber (2007) noted, Black men have been positioned as a threat to white womanhood, supporting the over-policing and criminalization of Black men and boys. In the words of Angela Y. Davis (1983) “fear of Black people is grounded in a fear of crime,” (p.65) a category Davis argues was constructed to regulate and suppress Black people and others who challenge white supremacy.

### Male Victim Denial/Recognition Dialectic

Tensions arise between the view of SV as fundamentally gendered and the view that gendered frameworks silence significant constituencies of victims/survivors (Malinen, 2014; 2018). Some researchers worry that studying male victimization can pander to men’s rights advocates (Maricourt & Burrell, 2022; Gotell & Dutton, 2016) while others suggest ignoring male victims reinforces patriarchal gender roles and male-specific rape myths (Javaid, 2016; Smith et al., 1988). Clearly male victims/survivors exist. One study found 51% of a sample of male students from a midwestern U.S. university reported sexual victimization since the age of 16 (Turchik, 2012). Another study, conducted at three southwestern U.S. universities, found 74% of Mexican male participants and 84% of Caucasian male participants had experienced sexual harassment. Among 284 US male high school and college students, 43% reported experiencing sexual coercion (French et al., 2014). A questionnaire administered to 507 men and 486 women in introductory psychology classes at a US university found males were more likely than females to experience unwanted sexual intercourse. For male participants, unwanted sex usually resulted from social pressures linked to masculine stereotypes (Muehlenhard & Cook, 1988).

Although most research on male sexual victimization is produced in the United States (Javaid, 2015; Stephenson et al., 2022), like SV against women, male victimization occurs in many (perhaps all) nations and cultures. A Canadian study based on 59 interviews with clients of two male survivor centres found 57 had experienced child sexual abuse and 10 had experienced adult sexual assault (McDonald & Tijerino, 2013). Another study, conducted in Ontario, Canada, collected data from 38 male clients of 29 hospital-based sexual assault centers over a year, further supporting the fact that male victimization happens. Researchers have addressed male victimization in South Africa (Mgolozeli & Duma, 2020), Sweden (Petersson & Plantin, 2019), and India (Das et al., 2022).

Perceptions of male victims/survivors and barriers to disclosure have also been explored. One US study found participants believed male survivors would encounter more barriers to disclosure and receive less help through university systems than female survivors. The authors suggest programming should “directly address the influence of gender on sexual assault disclosure and help seeking” (Allen et al., 2015, p. 109; see also Sorsoli et al., 2008; Brinkman et al., 2016; Haryanto, 2018). A second US study found “few positive experiences of disclosure” (Sorsoli et al., 2008, p. 339) among male victims/survivors as well as personal, relational, and sociocultural barriers to disclosure. A third found barriers to help-seeking for male victims/survivors including services not available to males, shame and embarrassment, denial, stigmatization, and fear (Tsui et al., 2010).

Despite having no questions about SV/SA experiences on our discussion guide, many female participants identified as victims/survivors. In contrast, although male participants likely included victims/survivors, none identified as such. As Weiss (2010) has put it, “when men report sexual victimization, they are publicly admitting that they were not interested in sex, were unable to control situations, and were not able to take care of matters themselves—all statements that run counter to hegemonic constructs of masculinity” (p. 293). Whereas sexual assault against women and girls can be understood as misogynistic enforcement of a socially constructed female place in society (Malinen, 2014; 2018), sexual assault against men or boys is often understood as injuring manhood/masculinity (Ralston, 2020; Sorsoli et al., 2008; Mgolozeli & Duma, 2020). Hegemonic masculine norms (Connell, 2005/2005) are enforced by peers and internalized by individuals, gatekeeping what men can express and how (Carlisle & Schmitz, 2022). One mode of gatekeeping is applying emasculating, misogynistic language to men who are victimized (Javaid, 2015). Another is labelling men sexually victimized in sport or fraternity settings as “hazees” (Malinen & Tobin, 2020; Fogel & Quinlan, 2021) or in war as “torture victims” (Card, 1997). With respect to male sexual victimization, Gunnarsson (2018) suggests dialectics may grasp the inter-implication of experience and discourse as well as the irreducibility of the former to the latter.

## Methodology

The CAPSAP study explored students’ perceptions of SV/SA policies and services to inform culturally responsive approaches in post-secondary institutions. Faculty from departments of Sociology/Anthropology, Education, Women and Gender Studies, Psychology, English Language and Culture, and Nursing formed the research team. Collaborators included a Harassment and Discrimination Officer and International Education Centre Manager. Other members included a student coordinator/research intern as well as paid peer facilitators. During Phase One, focus groups were in-person. Phase Two moved to a virtual format due to COVID-19 restrictions. Except for one focus group, participants and peer facilitators identified as belonging to the same gender and cultural region. The remaining group comprised transgender and non-binary participants from any cultural region.

Constructivist Grounded Theory Analysis (CGTA) was performed as follows: 1) Researchers from each institution coded their institution’s transcripts, by “comparing data with data to find similarities and differences” (Charmaz, 2014, p. 132) within focus groups, across the data set, and in relation to cultural and gender-based categories. Through constant comparison, we consolidated codes into themes, sometimes “enlisting” disciplinary terms (p. 133) like “dialectic.” Where problems appeared that could be addressed with concrete measures, we generated recommendations for institutional reports (Takševa, 2021; VanTassel, 2020; VanTassel et al., 2021). 2) The principal investigator and research intern consolidated and expanded institution-level themes and recommendations into an overarching analysis, which was reviewed and approved by co-researchers and collaborators. Team members continue to reanalyze qualitative data from diverse disciplinary and theoretical perspectives to illuminate participants’ contributions from multiple angles.

GTA is a dialectical method in that it is “iterative,” “inductive,” “comparative,” emergent, and open-ended” (Charmaz, 2014, p. 12). Our approach is more constructivist than positivist in that we understand our “data and analysis as created from shared experiences and relationships” among research team members, peer facilitators, and participants (p. 239). We examine the “interpretive work” (ibid.) of research participants and peer facilitators. We understand this work as having unfolded in layered contexts including ideas about political correctness, culturally shaped gender norms, and immigration/visa status.

Perspectives of seven cultural communities included in our study appear in the current article (African Nova Scotian, Canadian, North African, North Indian, South Asian, South Indian, West African) while seven communities included do not appear in what follows (Caribbean, Bermuda, Bahamas; Chinese; East African; Latin American; Southeast Asian; Turkish and Middle Eastern). No data was collected from male students originating from the Caribbean, Bermuda, Bahamas; East Africa; Latin America; or Southeast Asia due to lack of peer facilitators and/or participants. At each university, recruitment aimed for nationally or regionally delineated cultural categories large enough to support a five to seven person focus group per gender. Focus group delineations varied among universities and between national and regional compositions. For example, we held both South Indian and North Indian focus groups at two institutions. Fewer students from West African or Latin American countries meant focus groups were held for each region as a whole. African Nova Scotian focus groups were also held following encouragement from community members in recognition of this community’s distinct history, including over-policing. African Nova Scotians may also have participated in Canadian focus groups, although none identified themselves as such. Our focus group method, including implications of using countries as proxies for culture (Moon, 2013; Ono, 2013) is explored in detail elsewhere (Malinen, 2023).

Discussions began with presentations summarizing SV/SA policies. Focus group interviews covered 1) participants’ questions; 2) pre-existing familiarity with policy; 3) perceived policy relevance; 4) elder family members’ views on the policy as imagined by participants; 5) supporting a friend following disclosure; 6) supporter qualities and characteristics; and 7) advice to cultural outsiders about supporting insider victims/survivors. Facilitators were encouraged to follow topical threads as they arose.

The interview guide included no questions about wrongful allegations, respondent treatment, or male survivors. Thus, repeated emergence of these topics suggested their importance to students. Themes unforeseen in creation of the research instrument would not likely have emerged had we used a quantitative approach with closed-ended questions. Certainly, the nuance and tension that surfaced in discussion between participants would not have found an outlet. One question asked participants to imagine a friend had disclosed SA. The pronoun “she” was chosen to refer to this hypothetical friend on the premise it would evoke a more universally relatable example since women are statistically more likely than men to be sexually assaulted whereas men are statistically more likely than women to be perpetrators. In practice, many facilitators substituted “he or she” or “they,” producing a gender-neutral alternative. In retrospect, more than enhancing relatability, the female pronoun may have upheld denial of male victimization. This is an alarming thought given that data on male victimization presented in our literature review shows male victims/survivors are common and no doubt numbered among participants, although none self-identified. That women are more likely than men to be victims/survivors does not reduce the trauma that male victims/survivors experience. Where male peer facilitators followed the discussion guide in gendering the hypothetical survivor female, current or future victims/survivors participating may have felt invisible. This may have produced regrettable consequences for future willingness to engage needed supports.

## Dialectical Themes

The passages below exemplify tension among anxieties about wrongful accusations, opposition to victim-blaming, and perceptions that, for men, disclosure avenues are closed. In several focus groups, voicelessness was linked to racism and/or ethnocentrism.

### Wrongful Blame Dialectic Exemplar passages

Whereas approximately two-thirds of focus groups were comprised of female students, approximately one-third of transcript segments coded as “concerns about respondent rights” were contributed by women. These concerns were emphasized primarily by male participants, as exemplified in the following passages drawn from Canadian, African Nova Scotian, West African, and North African focus groups. Racial or ethnic bias is raised by members of all but the Canadian group, which may have included only white students.

#### Canadian

The first exemplar passage categorized under the Wrongful Blame Dialectic is extracted from conversation among Canadian male participants. This discussion concentrates at the malicious accusation pole of the Wrongful Blame Dialectic, with participants discussing feelings of vulnerability to false allegations. From a dialectical perspective, fear of being wrongfully blamed for SA/SV as a male can create a tension between acknowledging the gravity of the issue and empathizing with victims/survivors while also safeguarding against false allegations. Both perspectives can coexist and require careful consideration.

**Participant One:** In the actual definition base, I’ll take issue with the immediate designation of those reporting assault as a “survivor.” Er, from a legal standpoint, that—just, just from a language standpoint, it’s not even a definition standpoint—immediately shifts the burden of proof to the respondent. It makes the assumption that the individual, er—without, from my perspective, without actually diminishing the intensity of, er, sexual abuse and sexual assault, nor the legitimacy of actual survivors—but in the abstract word, for people who abuse this system, er, it is a window through which to do this. For example, if I were to immediately at this very moment falsify a claim against you, saying you had sexually assaulted me, I would instantaneously be labeled as a “survivor,” which language-wise, immediately puts a noose surrounding your neck that you have to try to remove instead of the standard prosecutor-defendant relationship.

[…]

**Participant Two:** I’d like to know why [the policy] doesn’t touch on false allegations, or whatever. As we mentioned previously, like, our demographic more typically is um, being a respondent role. So, as a personal, like, fear, I’m more afraid of being falsely—like, having false allegations against me than actually, um, being harassed.

[…]

**Facilitator:** So maybe I can ask this: Has anyone had any experience of false allegations, or? I don’t mean you personally, but even has it ever been a concern for you or anyone you know, about false allegations?

[…]

**Participant Three:** It’s er, along the lines of a, a passive fear. The same fear that, when I cross the street: I make the assumption that I’m not going to get hit by a car, but the chance is still there. I still have to take that into account when I cross the street.

**Facilitator:** Yeah, I see, yeah […]. Participant Four, go ahead.

**Participant Four:** Yeah, er, like he said, it’s kind of like, subconscious fear that’s around now and it’s due to kind of, the climate—

**Facilitator:** Yeah.

**Participant Four:** —recently, I guess, you could say. Because there has been like, er, a record number of false claims over the past five years, fair to say.

Canadian Participant One highlights the seriousness of SV/SA experienced by “real survivors” while expressing fear of wrongful allegations. Affirming the seriousness of SV/SA may simultaneously forestall risk of judgement associated with expressing a politically fraught view (McKendy, 2006) and express a genuine belief. Perhaps gaining confidence from hearing their peers, Participants Two, Three, and Four do not follow suit, focusing fully on wrongful accusations.

Participant One metaphorizes immediate use of the term “survivor” with the image of a noose placed around the neck of someone who may be innocent. This metaphor suggests danger, fear, helplessness, injustice, and existential threat. Similarly, Participant Three describes his fear of false allegations as a “passive fear.” While not applicable in the technical sense (Hatzimoysis, 2014), this term evokes danger, helplessness, and innocence. These implications are reiterated by the “crossing the street” metaphor, which additionally suggests activities anteceding wrongful accusations are normal, inoffensive, and pervasive.

As a man, Participant Two feels at risk for wrongful allegations but not SV. His perspective is the reverse of research findings that tell us a significant portion of male university students experience SV/SA, while a small percentage of a small number of complaints are false. This reversal could be read as cynical gender-political manipulation, however a simpler explanation is that reports of wrongful allegations but not male victimization circulate among male students. For instance, Participant Four could not have known the number of false claims made over time (whether in his university or a broader arena) to establish that the most recent 5 years were record-setting. However, he may have been aware of more than one accusation that he understood as false. Either way, Participant Four’s comments exemplify speech that may fuel fears among male students. Participants in this focus group may have left more concerned about wrongful accusations than they were on arrival.

#### African Nova Scotian

Like Participant Two in the Canadian male focus group, African Nova Scotian Participant One saw lack of consequences for wrongful allegations as a source of injustice. From this African Nova Scotian participant’s perspective, however, deterring or punishing wrongful allegations was a matter of racial equity, illuminating the complexity of addressing SA/SV and the intersectionality of factors such as race and gender.

**Participant One:** It [the policy] doesn’t really explain how misuse of the policy, like if somebody was to be wrongfully accused— and the—where I'm going with this is I feel like—and I can’t really reference any specific data on this—but maybe African Nova Scotian males have been, I guess, more—more targeted or more disadvantaged by the enforcement of this policy than any other demographic. And for that reason, it doesn't really give, you know, any clear consequences as to somebody who decides to enforce this—enforce maybe some of the measures of these policies which have like, everlasting consequences, obviously.

Although Participant One stated a lack of supporting data for his view, ample evidence suggests disproportionate policing of Black men in colonial societies including Nova Scotia. This focus group closely followed a report confirming what African Nova Scotians already knew: that police street checks in the area have criminalized young Black men and undermined trust in the criminal justice system (Wortley, 2019). Distrust is exemplified by Participant One’s sense that his peer group was “more disadvantaged by the enforcement of this policy than any other demographic.” Like other male participants, Participant One saw (wrongful) enforcement of policy measures as having lifelong consequences. Danger, fear, helplessness, and persecution are communicated by terms like “targeted” and “disadvantaged,” in contrast to the empowerment suggested by the idea that complainants can “decide to enforce” policy measures.

Although racial bias and wrongful allegations were a primary focus of African Nova Scotian male participants, this focus group discussion later included recognition of the importance of “believing women”:

**Participant Three:** I think it’s so important that we believe women when they come forward, so that way when women, you know, they have these experiences, they can come forward and not be criticized, etc. So, it’s so important to believe them. But I think, the other side is, I just think that just being, you know, educated on—you know, on how there’s so much overrepresentation in, you know, the legal system and things like that. I just feel like, you know, if I—if I was to have an allegation against me, then I feel like I'm already one step behind being Black, as people have perceptions of others. So, I’m just in the same boat as everyone else, I think.

As with Canadian Participant One, the reference to believing survivors may simultaneously express African Nova Scotian Participant Three’s values and provide cover for the more politically risky suggestion that wrongful allegations occur, although Participant Three was not the first in his group to introduce the latter topic. The fact that the term “women” takes the position of “victims/survivors” in this passage reiterates prevailing gendered views. Also notable is Participant Three’s dialectical presentation of “believing women” as one side of the issue and of “understanding overrepresentation of Black men” as the other side.

#### West African

As with Canadian and African Nova Scotian male participants, West African male participants readily imagined implications of false allegations for respondents. Here, the focus is on interim measures, such as barring a respondent from a residence or class, that can be implemented, as deemed necessary in the interest of safety, before an investigation concludes. Participants in this focus group worried their demographic might receive inequitable treatment based not only on gender, but also on race or nationality.

**Participant One**: I’d like to know why the accused person is treated as if they're guilty of the offense even when they—they weren't through with investigations. I want to know why. […]

**Facilitator:** So, what's the question? […]

**Participant One:** The question is why is the accused—

**Facilitator:** Guilty until proven innocent, or innocent until proven guilty?

**Participant One:** Yeah, yeah. No, obviously not innocent until proven guilty because, there's restrictions on—. You see they said the—the accused is not allowed to go to classes and stuff, right? So, stuff like that. […] So, say you're—you're innocent and you're being deprived of going to your classes that you've paid for. That's an inconvenience they’re never going to give back to you. You know? They cannot restore that to you. You know what I'm saying?

Like African Nova Scotian Participant One, West African Participant One cited irrevocable consequences of wrongful allegations. While anxieties about bias against West African men are not raised in the foregoing transcript passage, they did appear later in the focus group conversation:

**Facilitator:** What advice would you give to the security personnel, Guidance and Counseling Service, Office of Student Experience, the President? What advice would you give to them [about] students from Ghana or West Africa [who] have been sexually assaulted, because—

**Participant Two:** Handle everybody with care.

**Participant One:** And equality.

**Participant Two:** And equality, you’re right. […] Treat me like how you would treat a white. Like a citizen of Canada. Treat me with equality. […] It’s not like because you're from West Africa, or we are, we don’t know what is right or something. So, because of that, we are from—we are from Ghana, or something, so because of that we can look at a woman and then call inappropriate names or slap her ass or do anything we want.

**Facilitator:** Okay.

**Participant Two:** OK? If the question is asking that, because you are from Western Africa, or—we are like that, then it really gets me mad. If that’s what that question is saying.

**Facilitator:** No, the question is saying what advice would you give to them? Should they see you, oh, “Because I'm from West Africa—”

**Participant One:** See? So, that's what he said. Treat me as a human being. Treat me as a reasonable human being, as I come, as you see me. You see me as a sane human being, visibly. So, treat me as you treat any person who is involved in any assault case.

Perhaps because West African Participants One and Two were thinking through the lenses of their own positionalities, which they did not perceive to include potential victimhood but did understand to include persecution, when asked about advice for supporting victims/survivors from their region, they replied with remarks on respondent treatment. Their objections may have been articulated in part against the background of internationally mediatized SV within West African universities and high schools, a reality that could fuel stereotypes to the detriment of West African respondents. Female participants from the region spoke about victim-blaming of women, commonness of SV, and a pattern of quid pro quo harassment by male professors against female students. This pattern has also been explored by researchers (Morley, 2011; Norman et al., 2013; Frank, 2019; Steiner & Spear, 2020).

In calling for equal treatment, Participants One and Two alternately stated that West Africans should be treated as “whites,” “Canadians,” or “human beings.” As stated by Maynard (2017), “tropes of anti-Blackness that were created centuries ago are reproduced within the racialized surveillance and punishment of Black migrant and racialized communities” (p. 161) in Canada. In short, West African male Participants One and Two perceived that white and Canadian respondents would be treated as humans, whereas Black or West African respondents might not.

#### North African

The final exemplar passage for this section is drawn from the North African male focus group. This conversation focuses on problematizing victim-blaming, more briefly addressing bias as it relates to potential false complaints against the group’s demographic. It also exemplifies tension between idealization of Canada and more balanced assessment of Canadian culture as it relates to victim blaming:

**Participant Two:** I think I’d agree with Participant Number One from the previous question that unfortunately our culture sometimes has the tendency to blame the victim, uh, saying that it’s the dressing. Probably the biggest thing they say is, “You were dressing too inappropriately, so you were asking for it.” That’s always the sentence that we hear about. So, I think as someone that has come to a more western culture, of course, in Canada […] this type of thing is never blamed on the victim, which is a good thing, of course. And you realize that there’s more help advertised everywhere than back home.

**Facilitator:** Um, the other participants?

**Participant One:** I don’t know if this is relevant or not, but there’s this stereotype about North Africans or Arabs that they are controlling, which sometimes, in some situations—I’ve even seen it—it will be easier to violate this policy by just [saying] that “This person from North Africa” or “This Middle Eastern person has sexually assaulted me.” It’s way easier for them to be trusted just based on the stereotype that they are more controlling; they are more violent.

**Facilitator:** Yes, Participant Number Three, please go ahead.

**Participant Three:** I think even though in the western culture it’s way more accepted to talk about these things than in North Africa, I still think it’s not enough and we still need—we still have some work to do. We need to be more supportive of these topics. And another thing is, usually, if a man sexually assaults a woman, the blame, as the other participant said, the blame goes to the woman more than the man. And that’s completely wrong, and the man is the one that should be blamed one hundred percent. Whether the woman was dressed provocatively or not, that should be irrelevant.

Participants’ assessment that victim-blaming is common in North Africa is echoed by research. For example, according to a 2008 study by Boy and Kulczycki, in the Middle East and North Africa, it is commonly believed that husbands should beat wives who refuse sexual intercourse. On the other hand, North African participants’ vocal opposition to victim-blaming serves to counteract stereotyping. Participant One occupied the tension between the poles of the Wrongful Blame dialectic. He was first to problematize victim-blaming, as referenced by Participant Two at the beginning of the above passage yet said he had observed bias against Middle Eastern and North African male respondents. Participant Three re-centres problematization of victim-blaming, articulating this issue in gendered terms: “usually the blame goes to the woman,” whereas “the man is the one that should be blamed, one hundred percent.”

The above passage is also characterized by dialectical interaction between the idealization, expressed by Participant Two, that victims are never blamed in Canada, and Participant Three’s more realistic assessment that more support for victims/survivors is needed in both Western and North African contexts. Crucially, higher levels of gender-based violence and victim-blaming in a region; resulting elevated awareness of these problems on the part of individuals from that region, including men; and bias against these same individuals are not mutually exclusive.

### Male Victim Denial/Recognition Dialectic Exemplar passages

As Javaid (2015) notes, “men respond to and define situations according to the anticipated or imagined reactions from societies and the reflected appraisals received from other men during social interaction” (p. 14). Canadian, South Indian, African Nova Scotian, and South Asian male focus group passages illuminate “the interplay of expectations about gender roles and disclosure processes, including the decision to disclose or not” (Sorsoli et al., 2008, p. 344). This section reflects internal and external forces militating against male reporting or disclosure. It captures gendered performances that communicate expectations for masculinity.

#### Canadian

Drawn from the Canadian male focus group, the following passage exemplifies strong reluctance to believe that male students can be vulnerable to sexual victimization – a reluctance regarding which the speaker displayed high self-awareness.

**Unknown Participant:** Following the stigma category of sexual harassment—men not […] being harassed or not qualifying—I immediately probably wouldn’t completely believe one of my male friends [if he told me he had been sexually assaulted]. I would think it was more of a sick joke they are trying to make, say, compared to a female friend. I would believe them without doubt. However, in that situation, I would […] question more to find out what’s going on, and if it, like, actually was a sexual harassment occurrence, I would try to coach them towards disclosing the information at the minimum and be there for them if they need support.

Clearly the Unknown Canadian Participant cited above believes women, not men, are subjected to SV/SA. He gatekeeps the category of victim/survivor along gendered lines. He suggests that men are rarely if ever victimized; that any suggestion of male victimization is likely a joke; if it isn’t a joke, it’s probably a misunderstanding; and if it’s neither a joke nor a misunderstanding, it likely had little or no negative impact. Thus, the person who has had the experience is not really a victim, would not require support, and might as well remain silent. To determine whether a sexual assault had truly occurred, the Unknown Participant would ask questions, and if he were to determine it had occurred, it would remain to be established if any support should be accessed. This approach is notably distinct from the unreserved belief the participant states he would extend to a female friend, not to mention more likely to cause distress (Evans & Coccoma, 2014).

The contrast between the blanket trust this participant claims in female friends’ disclosure and the perception, expressed earlier in the same focus group, that wrongful allegations are common, is also notable. One interpretation is that activating the imagined position of respondent may lead a man to defensively conjure the perspective of women as false complainants; whereas activating the imagined position of victim may lead a man to defensively conjure the perspective of women (but not men) as vulnerable. Since we do not know which participant contributed this comment, it is also possible the speaker simply disagrees with the ideas expressed earlier. Alternatively, the Unknown Speaker may trust his friends, but not women in general.

#### South Indian

Whereas the Unknown Canadian Participant perceived male victimization as highly unlikely, what was unthinkable for South Indian Participant One was reporting such an event.

**Participant One:** For example, if you ask all the six or seven people in this group if something happened, then they’d report it to the cops? Or, for example, if a woman did something, then they’d report it or they’d keep it to themselves, [they’d say], “Oh, nothing happened.”

**Facilitator:** Okay, so, everybody, like, if something happens to er, any one of us in the group, like, they—we won’t report it, right? That’s what you are saying.

**Participant One:** Yes, exactly.

In confidently stating all focus group members would maintain silence and deny having been victimized if asked, Participant One communicated accepted standards for masculinity that forestall “subversion of the existing hegemony” (Bird, 1996, p. 122). However, we cannot know what personal experiences undergirded this (or any) statement. The statement could have been rooted in a general understanding of how masculinity works or could have been informed by personal experience of victimization and subsequent silence. Indeed, confidently stating that nobody sharing one’s demographic position would disclose may be the nearest some people get to disclosure.

#### African Nova Scotian

Conversation among African Nova Scotian participants suggested men’s decisions to remain silent rather than seek support may be overdetermined, namely by the perception they would be disbelieved due to overlapping identity factors and the risk of shame accompanying disclosure.

**Facilitator:** If something happened with, like, anyone here, or like, do you guys think—would you—would you be comfortable going forward with the allegation based on the policy, and through that whole process, like, especially as Black men? Like, is that something that we would comfortable doing?

**Unknown Participant:** Yeah, I personally wouldn’t feel comfortable just because I know that, you know, my word would be questioned. And, you know, I feel like even coming to them with the—with the concern of something happening, they’re not really going to take it at face value because, you know, they’d—they’ll take a look at me and see that, you know, I'm a taller you know, Black man. So, it’s just something that, like, I personally wouldn’t feel comfortable with.

[…]

**Participant One:** Like um, you know, I would—I feel like I wouldn't have a voice at all in this process. […] So, I wouldn’t honestly—say, if this happened to me, I would just crawl into a hole and just hope for the best that, ah, you know, I wouldn’t—I wouldn't know who to turn to, based on what I’m seeing here. You know, what my rights are? Do I have a voice? Are people even going to respect what I’m saying if I were to come forward? That isn’t what happens. […] That’s just how I feel about these processes and policies is just, you know, avoid at all costs, as a Black man.

[…]

**Facilitator:** Sure. I mean, maybe even another question I would ask, like, you guys, like, […] how would you guys feel, like, if you had to go forward with something like this and, like, your friends started finding out when people started to talk about it? Like, I would—how would that make you guys feel? […]

**Participant Two:** If I had to go through with something like this, I'd probably definitely feel embarrassed and, you know, not want to see anybody. You know, my mental health would be shot. […] I’d have to—I would have to confine myself to my house. I feel like I couldn't face anyone if I had to go through something like that.

**Participant Three:** Yeah, I agree with everything you just said. And I wouldn’t be able to go out of the house, and ah, I would probably delete my social media stuff, and yeah, your mental health would be definitely shot, and nowhere really to turn to at that point. I don't know, what could you really do?

For participants in this group, the expectation that a disclosure of SV would not be believed, seen, or heard manifests in phrases such as “They’re not going to take it at face value,” and “I wouldn’t have a voice.” This expectation is intersectionally constructed in terms of appearance, for example in the Unknown Participant’s explanation that he is “a taller Black man.” Participants Two and Three imagined that if they were sexually assaulted and this fact became known, they would attempt to disappear: “I would have to confine myself to my house,” “I couldn’t face anyone,” “I would delete my social media stuff.” Between appearing as an ineligible victim, not being taken at face value, and wanting to disappear, in the words of Participant Three, there would be “nowhere to turn.”

#### South Asian Male

By citing an example of male victims/survivors, in this final exemplar passage, South Asian Participant One positioned himself as an outlier among male participants who was prepared to challenge hegemonic masculinity.

**Participant One:** A lot of people, they think that male genders, they don’t experience something like that. When I was on work one week I actually [encountered] an incident like that. It was not on campus; it was off campus, because I was working for a volunteering association […]. Male genders, they do experience it, but are not able to express it ’cause of the stereotypes around masculinity.

Not only did this participant directly state that male victimization occurs, he also challenged lack of recognition, attributing the silence he was breaking to gender-based stereotypes.

## Recommendations and Conclusion

Most SV/SA interventions are reactive and women-focused, without proactive strategies aimed at changing the socialization of boys and men. Intrinsic to hegemonic masculinity (Connell, 2012) is the repudiation of the feminine and the validation of male power. Sexual violence perpetrated by men towards women is often a form of recuperating power to which the perpetrator feels entitled. Concomitantly, experiences of unwanted sexual contact can undermine one’s sense of masculinity. Yet masculinity is dynamic, changing across time, shaped by culture, politics, and society (Toledo del Cerro, 2022). Nonviolent masculinities also exist across cultures. Consider the North African Male participants who vocally opposed victim blaming in North Africa and beyond. They produced dialectical tension relative to hegemonic maleness in the form of “caring masculinity” (Connell, 2012) that rejects dominance.

In both themes explored above, participants expressed powerlessness. According to a certain reading, these voices would be interpreted as a cynical sleight of hand that hides patriarchal power from those who might otherwise help dismantle it. However, this interpretation arguably manifests a silencing “mechanism of oppression” (Martinez, 2006, p. 305). Whatever the actual risks, male participants felt more vulnerable to wrongful accusation than to SV. Male participants also felt they were more likely than women to be blamed and disbelieved, whether as respondents or as complainants. These findings have important implications for creation, provision, and promotion of SV/SA policies and service provision.

When questions arise about wrongful accusations, we often suggest such situations are too rare to consider. Indeed, formal accusations are relatively rare, and only a small minority of these seem to be false. However, such responses do not quell the anxieties of students who raise the question. People largely do not experience their worlds in terms of statistical probabilities and may not trust the statistics they are given. Furthermore, we have shown that some anxieties concerning wrongful accusations are shaped by awareness of ethnic and/or racist stereotypes. African Nova Scotian participants who problematized victim-blaming of women mirrored the double bind faced by women of colour between the imperatives of addressing intra-community violence against women and racist persecution of men (Crenshaw, 1991).

Communicating about investigations in a way that demonstrates respondents are not automatically judged guilty would provide important information for students who have experienced SV and assuage anxieties of those who feel vulnerable to wrongful accusations. Using the word “complainant” rather than “survivor” in investigative contexts, as some universities do, could make a difference.

Providing clarity about national laws and campus policies is also important. International students may worry about crossing legal or policy boundaries that do not exist at home. As one South Asian male put it, “A lot of different things […] might be a sexual violence for someone else coming from a different culture, and it contradicts a lot with the policies, which I think Participants Four and Five said. There should be more educational resources, not just on one sexual policy, but it should try to include different cultures as well.”

While continuing to problematize the normalization of violence against women, we can also highlight data showing male sexual victimization is a hidden reality in cultures worldwide, including within western universities. Raising awareness of male victimization statistics may help male victims/survivors to find support rather than incredulity or ridicule among their peers and to feel more confident reaching out to formal care providers. Whereas male students may be less likely than female students to initiate investigations, efforts should be made to increase the number of male care providers, preferably from diverse cultures. To this end, universities can partner with online services that offer counselling with professionals worldwide and/or with community organizations.

Thinking dialectically about concerns regarding wrongful blame, victim-blaming, and victimization across genders would involve continuing to counter hegemonic masculinity, as well as ethnic and racist stereotypes, communicating that all students are more at risk of SV than of wrongful accusations, and attending to respondent rights that may already be integral to policies yet underemphasized in outreach activities. Such an approach may increase awareness, trust, and engagement across the student body, leaving more campus community members (rightly, we hope) with the impression that justice is possible.
